# Adiposity and risk of oesophageal cancer subtypes in the Million Women Study

**DOI:** 10.1093/ije/dyad094

**Published:** 2023-07-12

**Authors:** Siân Sweetland, Sarah Floud, Kezia Gaitskell, Gillian K Reeves, the Million Women Study Collaborators, Emily Banks, Valerie Beral, Lucy Carpenter, Carol Dezateux (chair), Sarah Floud, Jane Green, Julietta Patnick, Richard Peto, Gillian Reeves, Cathie Sudlow, Simon Abbott, Rupert Alison, Sarah Atkinson, Krys Baker, Angela Balkwill, Isobel Barnes, Judith Black, Roger Blanks, Anna Brown, Andrew Chadwick, Dave Ewart, Sarah Floud, Kezia Gaitskell, Toral Gathani, Laura Gerrard, Adrian Goodill, Carol Hermon, Darren Hogg, Alison Hudak, Isobel Lingard, Sau Wan Kan, Nicky Langston, Keren Pepier, Kirstin Pirie, Gillian Reeves, Keith Shaw, Emma Sherman, Karl Smith-Byrne, Helena Strange, Siân Sweetland, Ruth Travis, Lyndsey Trickett, Clare Wotton, Owen Yang, Heather Young

**Affiliations:** Cancer Epidemiology Unit, Nuffield Department of Population Health, University of Oxford, Oxford, UK; Cancer Epidemiology Unit, Nuffield Department of Population Health, University of Oxford, Oxford, UK; Cancer Epidemiology Unit, Nuffield Department of Population Health, University of Oxford, Oxford, UK; Cancer Epidemiology Unit, Nuffield Department of Population Health, University of Oxford, Oxford, UK

**Keywords:** Oesophageal cancer, body mass index, abdominal adiposity, squamous cell carcinoma, adenocarcinoma

## Abstract

**Background:**

The strong association of body mass index (BMI) with increased oesophageal adenocarcinoma risk is established, but its relationship with oesophageal squamous cell carcinoma is less clear. There is little evidence regarding the association of abdominal adiposity with either subtype.

**Methods:**

In a large prospective cohort of women in the UK, mean age 56.2 [standard deviation (SD) = 4.9] years, we investigated the risk of oesophageal adenocarcinoma and squamous cell carcinoma in relation to self-reported BMI, waist circumference (WC) and waist-hip ratio (WHR), using Cox regression to estimate adjusted relative risks (RR) and 95% confidence intervals (CIs), taking account of potential reverse causation bias.

**Results:**

During mean follow-up of 17.7 (SD = 4.9) years, 1386 adenocarcinomas and 1799 squamous cell carcinomas of the oesophagus were registered among 1 255 529 women. Compared with women of BMI 22.5 to <25 kg/m^2^, those with BMI ≥35 kg/m^2^ had a 2.5-fold risk of adenocarcinoma (adjusted RR = 2.46, 95% CI = 1.99–3.05) and an almost 70% reduction in risk of squamous cell carcinoma (RR = 0.32, 95% CI = 0.22–0.46). These associations were broadly similar in each 5-year follow-up period, and were evident in both never and ever smokers, although somewhat stronger for squamous cell carcinoma among current and past smokers than in never smokers (*P*_heterogeneity_ = 0.007). After controlling for BMI, WC and WHR were associated with risk of squamous cell carcinoma but not adenocarcinoma.

**Conclusions:**

In this population of middle-aged women, there was robust evidence that greater BMI is associated with an increased risk of oesophageal adenocarcinoma and a reduced risk of squamous cell carcinoma.

Key Messages

- In this large prospective study of body mass index (BMI) and oesophageal cancer subtypes in women in the UK, we assessed the degree to which observed relationships with each subtype may have been influenced by bias.
- The findings provide the most convincing evidence to date that greater BMI reduces the risk of squamous cell carcinoma of the oesophagus.
- After controlling for BMI, abdominal adiposity appeared to be independently associated with risk of squamous cell carcinoma but not adenocarcinoma.
- Further research into the mechanisms underlying the inverse association of adiposity with squamous cell carcinoma of the oesophagus is warranted.

## Introduction

Oesophageal cancer accounts for about 3% of cancers and about 6% of cancer deaths,^1^ worldwide. Almost 80% of oesophageal cancers worldwide occur in Asia,^1^ where squamous cell carcinomas account for the vast majority of cases (70–90%).^2^ In Western populations, overall incidence rates are lower^2^ and, whereas rates of squamous cell carcinoma have decreased over recent decades, rates of adenocarcinoma have increased,^3^ such that in 2019, adenocarcinomas accounted for about two-thirds of all oesophageal cancers in England.^4^

Given its relatively low incidence, most epidemiological studies of adiposity and oesophageal cancer have been retrospective and therefore prone to differential recall of exposures between individuals who have been diagnosed with cancer and those that have not. Case-control studies are also prone to reverse causation bias, whereby individuals with cancer experience weight loss prior to diagnosis due to conditions predisposing to oesophageal cancer or to the cancer itself. The few prospective studies that have examined adiposity in relation to risk of oesophageal cancer subtypes have tended to report positive associations between body mass index (BMI) and adenocarcinoma,^5–9^ whereas some have reported inverse associations with squamous cell carcinoma.^5^^–^^7^^,^^10^^–^^12^ There is little evidence as to whether associations with risk of oesophageal cancer differ by markers of body fat distribution. The causal relevance of the findings for squamous cell carcinoma remains unclear however, since associations based on prospective data, particularly in the short term, may still be subject to reverse causation bias. Previous studies have lacked sufficient case numbers and length of follow-up to reliably account for this potential bias.

We present findings from a cohort of 1.3 million middle-aged women in the UK, the Million Women Study,^13^ on the relationship between BMI and abdominal adiposity and risk of oesophageal cancer subtypes, taking careful account of potential reverse causation and confounding, particularly by smoking and alcohol consumption, which are less common in obese women and known to increase the overall risk of oesophageal cancer.^14^

## Methods

### Data collection, follow-up and definitions

In median year 1998 [interquartile range (IQR) 1998–99], the Million Women Study recruited 1.3 million women through the National Health Service (NHS) Breast Screening Programme.^13^ Women aged 50–64 [mean 56.2 years, standard deviation (SD) 4.9 years] were sent a recruitment questionnaire with their invitation to routine screening (asking about sociodemographic, anthropometric, lifestyle and health factors) and have since been resurveyed at approximately 3–5 year intervals. Women were followed up for deaths, cancer registrations and hospital admissions, via electronic linkage to routinely collected NHS data. Questionnaires and information on data access can be viewed at [http://www.millionwomenstudy.org]. All participants gave written consent for re-contact and for follow-up through screening clinic and other medical records.

Oesophageal cancer was coded as C15, using the International Classification of Diseases 10th Revision^15^ (ICD-10). Classification by subtype was based on the International Classification of Diseases for Oncology (ICD-O) morphology coding^16^^,^^17^ (Supplementary Methods, available as Supplementary data at *IJE* online).

In all analyses, BMI was calculated using self-reported weight at the relevant baseline survey and self-reported height at recruitment. Abdominal adiposity indices were based on waist and hip measurements first reported at the survey in median year 2001 (IQR 2000–03). Clinical measurements of anthropometric variables in a subsample of participants have been shown to be strongly correlated with corresponding self-reported measures.^18^

### Statistical analysis

The baseline for analyses of BMI and risk of oesophageal cancer subtypes was the recruitment questionnaire, completed in median year 1998. Women were excluded if they had any previous registration for malignant cancer (other than non-melanoma skin cancer, C44). Person-years were calculated from baseline to the first of: any malignant cancer (other than C44); death; emigration or other loss to follow-up; end of follow-up (31 December 2018).

We used Cox regression models, with time in study as the underlying time variable, to estimate hazard ratios (herein referred to as relative risks) and 95% confidence intervals (CIs) for adenocarcinoma and squamous cell carcinoma of the oesophagus by BMI (primarily categorized as <22.5, 22.5–<25 (reference), 25–<27.5, 27.5–<30, 30–<32.5, 32.5–<35, 35+ kg/m^2^). The proportional hazards assumption was assessed by use of tests based on Schoenfeld residuals; there was no evidence of a violation of this assumption. All statistical tests were two-sided.

Analyses were stratified by year of birth and year of recruitment, and adjusted for region, socioeconomic status, alcohol, smoking, oral contraceptive (OC) use, menopausal hormone therapy (MHT) use, menopausal status and age, strenuous exercise, parity and age at menarche (Supplementary Methods). Since the effect of smoking on squamous cell carcinoma has been shown to differ by alcohol intake,^19^^,^^20^ we fitted a combined variable for joint status with respect to fine categories of smoking and alcohol in the model. All adjustment variables included a separate category for missing data.

In analyses involving more than two exposure categories, results in tables and figures are reported in the form of group-specific confidence intervals, based on the variance of the log risk for each group,^21^^,^^22^ with conventional 95% CIs given in the text. Trends in risk per five-unit increment in BMI were corrected for measurement error by assigning a median measured BMI to each baseline category of self-reported BMI, using data from the substudy in 2008,^18^ and treating this variable as continuous (Supplementary Methods).

To investigate the potential impact of weight loss, caused by the cancer prior to diagnosis, on the association between BMI and oesophageal cancer, risk was examined in 5 year periods of follow-up time. Subsequent analyses excluded the first 5 years of follow-up to minimize any resulting impact.

The potential impact on the main findings of residual confounding by known risk factors was assessed by examining associations between BMI and oesophageal cancer by smoking status, alcohol intake and joint smoking and alcohol status, and by investigating the effect of individual adjustment for smoking and other potential confounders on the χ^2^ statistic relating to BMI (RR per 5  kg/m^2^) (Supplementary Methods). To assess potential interactions with other factors, we examined summary trends in risk per 5 kg/m^2^ increase in BMI in subgroups defined by eight other personal characteristics.

Sensitivity analyses investigated restricting adenocarcinoma diagnoses to ‘adenocarcinoma not otherwise specified’ and ‘adenocarcinoma of the gastro-oesophageal junction’ due to potential misclassification, and adjusting for self-reported evidence of gastro-oesophageal reflux (GORD) (Supplementary Methods).

The baseline for analyses of abdominal adiposity and oesophageal cancer risk was the survey in median year 2001. These analyses examined risk ≥5 years after this baseline, with the same censoring, stratification and adjustment criteria as described previously (Supplementary Methods). We initially compared RRs per 1-SD of BMI, waist circumference (WC) and waist-hip-ratio (WHR), using the median of the re-measured standardized values within each quartile; then we assessed the extent to which any associations of abdominal adiposity measures with risk were independent of the effect of overall adiposity (BMI) using the residual method^23^ (Supplementary Methods).

All analyses were performed using Stata version 17.1 (StataCorp, College Station, TX).

## Results

After excluding women with previous cancer (*n *=* *39 317), and those missing BMI at recruitment (*n *=* *69 422), there were 1 255 529 women in the main BMI analyses (see Supplementary Figure S1, available as Supplementary data at *IJE* online). During an average follow-up of 17.7 years, there were 1386 incident cases of adenocarcinoma and 1799 cases of squamous cell carcinoma. Obese women (BMI ≥30 kg/m^2^) were less likely to be smokers, drink alcohol, be past users of oral contraceptives, current users of MHT or do regular strenuous exercise, and had slightly lower socioeconomic status and earlier age at menarche, than women of BMI <25 kg/m^2^ (Table 1).

**Table 1.** Characteristics of the study population by BMI categories

Characteristic	BMI			All women	Women with adenocarcinoma	Women with squamous cell carcinoma
	<25 kg/m^2^	**25**–**<30 kg/m^2^**	30+ kg/m^2^			
*n* (%)	584 515 (46.6)	447 030 (35.6)	223 984 (17.8)	1 255 529 (100)	1386 (0.1)	1799 (0.1)
Age (years), mean (SD)	55.9 (4.9)	56.4 (4.9)	56.2 (4.8)	56.2 (4.9)	57.5 (5.0)	57.8 (5.0)
BMI ( kg/m^2^), mean (SD)	22.6 (1.7)	27.1 (1.4)	34.0 (3.8)	26.2 (4.7)	28.1 (5.2)	24.2 (4.0)
Highest socioeconomic quintile, *n* (%)	131 595 (22.7)	87 993 (19.8)	34 232 (15.4)	253 820 (20.4)	220 (15.9)	364 (20.4)
Current smoker, *n* (%)	125 284 (21.6)	80 606 (18.2)	34 557 (15.5)	240 447 (19.3)	413 (31.4)	607 (39.0)
Alcohol (drinks per week) among drinkers, mean (SD)	6.6 (5.5)	6.1 (5.4)	5.5 (5.3)	6.3 (5.5)	5.7 (5.4)	8.2 (7.1)
Non-drinkers, *n* (%)	179 910 (31.0)	157 245 (35.4)	103 420 (46.6)	440 575 (35.3)	599 (43.8)	665 (37.2)
Ever used OC, *n* (%)	357 775 (61.8)	257 832 (58.3)	123 540 (55.9)	739 147 (59.5)	718 (52.4)	946 (53.2)
Current MHT use, *n* (%)	210 572 (36.4)	145 065 (32.8)	61 747 (27.9)	417 384 (33.6)	380 (27.7)	507 (28.6)
Strenuous exercise at least twice per week, *n* (%)	142 337 (25.2)	83 642 (19.4)	30 334 (14.1)	256 313 (21.1)	224 (16.8)	385 (22.3)
Parity, mean (SD)	2.1 (1.2)	2.2 (1.2)	2.4 (1.4)	2.2 (1.2)	2.3 (1.4)	2.1 (1.3)
Age at menarche (years), mean (SD)	13.2 (1.6)	12.9 (1.6)	12.6 (1.6)	13.0 (1.6)	12.8 (1.7)	13.1 (1.6)
Postmenopausal, *n* (%)	414 995 (84.7)	328 034 (86.3)	160 825 (84.6)	903 854 (85.2)	1110 (89.9)	1521 (94.0)
Oesophageal cancers, *n* (%)						
Adenocarcinoma	435 (0.07)	527 (0.1)	424 (0.2)	1386 (0.1)		
Squamous cell carcinoma	1151 (0.2)	516 (0.1)	132 (0.06)	1799 (0.1)		
Other	190 (0.03)	125 (0.03)	71 (0.03)	386 (0.03)		

BMI, body mass index; OC, oral contraceptive; MHT, menopausal hormone therapy; SD, standard deviation.

**Figure 1.**
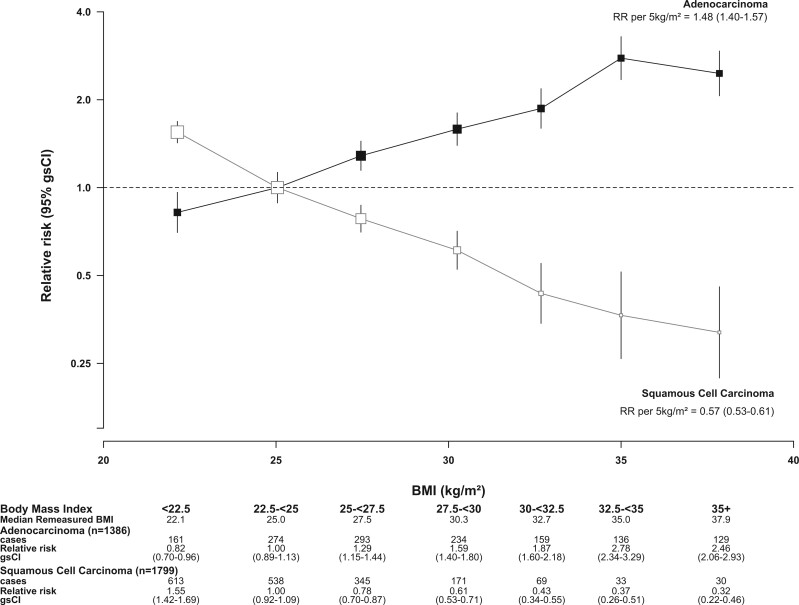
Relative risk of adenocarcinoma and squamous cell carcinoma of the oesophagus by BMI in all women. RR stratified by year of birth and year of recruitment; adjusted for joint smoking and alcohol status, OC use, MHT use, exercise, parity, menopausal status and age, age at menarche, region and socioeconomic status. RR, relative risk; (gs)CI, (group-specific) confidence interval; BMI, body mass index; OC, oral contraceptive; MHT, menopausal hormone therapy

There was a clear increase in the risk of oesophageal adenocarcinoma with increasing BMI (Figure 1), with a 2.5-fold risk for women of BMI ≥35 kg/m^2^ compared with women of BMI 22.5–<25 kg/m^2^ (RR = 2.46, 95% CI = 1.99–3.05). In contrast, oesophageal squamous cell carcinoma risk decreased substantially with increasing BMI, such that women with BMI ≥35 kg/m^2^ had a 68% reduction in risk (RR = 0.32, 95% CI = 0.22–0.46, versus BMI 22.5–<25 kg/m^2^).

**Figure 2.**
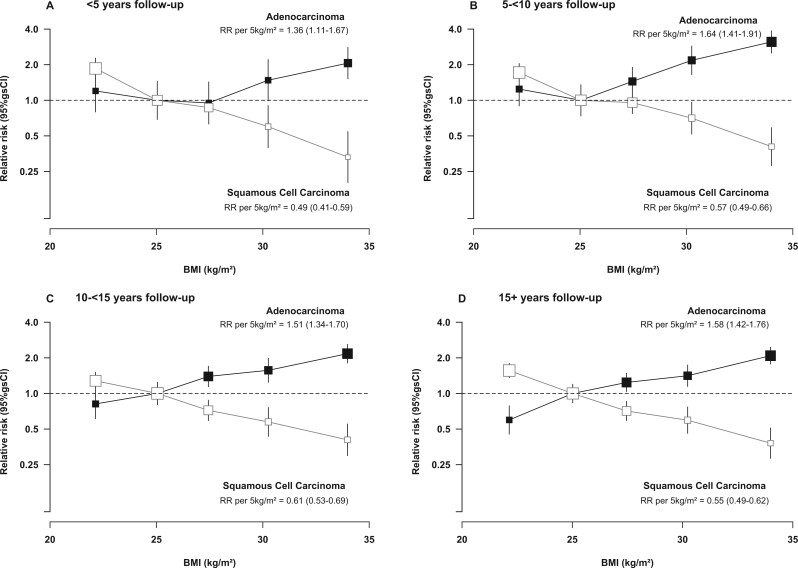
Relative risk of adenocarcinoma and squamous cell carcinoma of the oesophagus by BMI, in different follow-up periods. RR stratified by year of birth and year of recruitment; adjusted for joint smoking and alcohol status, OC use, MHT use, exercise, parity, menopausal status and age, age at menarche, region and socioeconomic status. RR, relative risk; g(s)CI, (group-specific) confidence interval; BMI, body mass index; OC, oral contraceptive; MHT, menopausal hormone therapy

There was a slightly smaller positive association of BMI with adenocarcinoma risk, and a slightly greater inverse association with squamous cell carcinoma risk, in the first 5 years (RRs per 5 kg/m^2^ = 1.36 and 0.49, respectively) compared with each of the subsequent follow-up periods (RRs = 1.64, 1.51, 1.58 and 0.57, 0.61, 0.55, respectively) (Figure 2). To minimize any potential impact of reverse causation on the short-term findings, subsequent analyses focused on associations with risk in the period 5 or more years after baseline.

**Figure 3.**
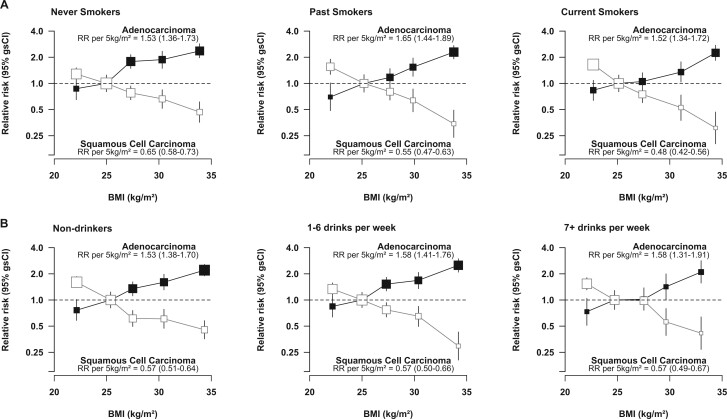
Relative risk of adenocarcinoma and squamous cell carcinoma of the oesophagus by BMI, in (a) never, past and current smokers and (b) non-drinkers, and women who drank 1–6 and 7+ drinks per week, excluding the first 5 years of follow-up. RR stratified by year of birth and year of recruitment and adjusted for OC use, MHT use, exercise, parity, menopausal status and age, age at menarche, region and socioeconomic status; results in (a) are additionally adjusted for alcohol intake, and in the case of current smokers for joint number of cigarettes smoked per day and alcohol status; results in (b) are additionally adjusted for smoking status and number of cigarettes smoked in current smokers. RR, relative risk; (gs)CI, (group-specific) confidence interval; BMI, body mass index; OC, oral contraceptive; MHT, menopausal hormone therapy

Similar positive and inverse associations of BMI with oesophageal adenocarcinoma and squamous cell carcinoma, respectively, were observed in women with different smoking habits (Figure 3a) and different alcohol intakes (Figure 3b), although there was a greater inverse association with squamous cell carcinoma in current and past than in never smokers (*P*_heterogeneity_ = 0.007). Strong associations were also evident in subgroups of women categorized according to both smoking and alcohol intake (Supplementary Figure S2, available as Supplementary data at *IJE* online). In further assessments of the impact of residual confounding by these and other factors, adjustment for smoking and alcohol had the biggest impact on estimated RRs per 5 kg/m^2^, but simultaneous adjustment for all factors considered had a relatively modest effect on the χ^2^ values associated with the BMI association (the χ^2^ value was reduced by 9.4% for adenocarcinoma and by 16.2% for squamous cell carcinoma after adjustment for all potential confounders) (Supplementary Table S1, available as Supplementary data at *IJE* online), indicating that the observed associations are unlikely to be unduly influenced by residual confounding by known confounders.

**Figure 4.**
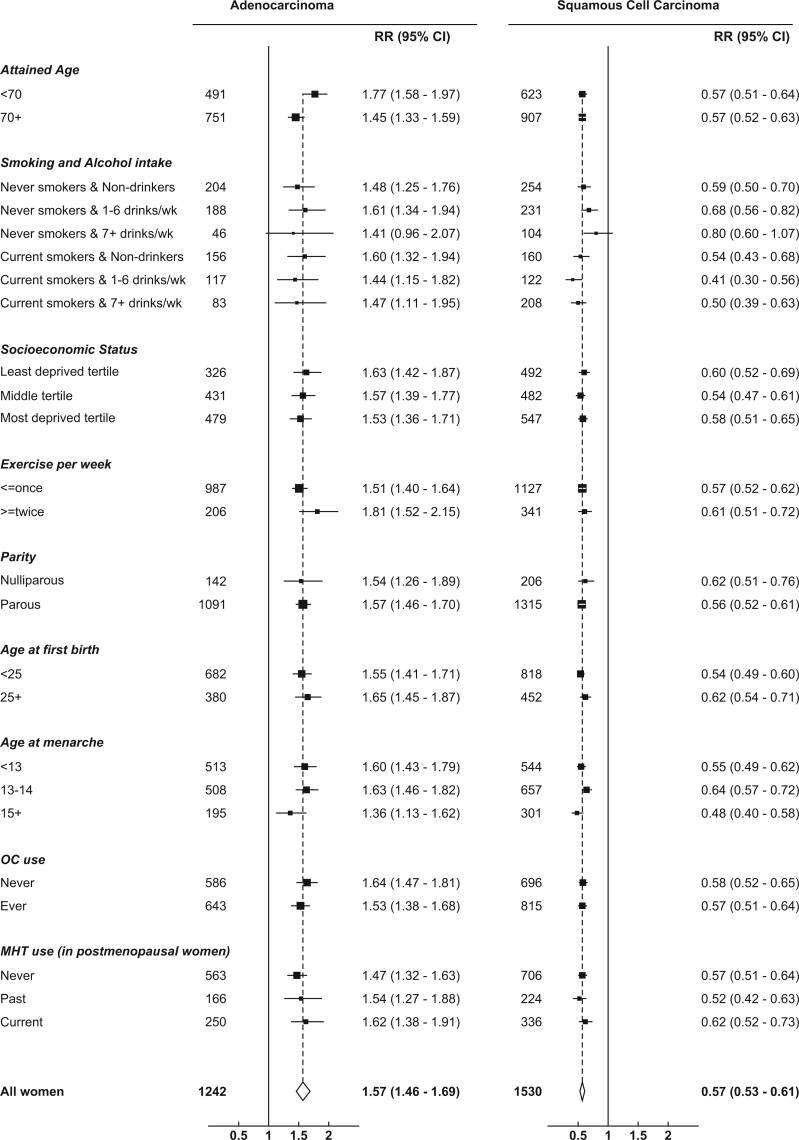
Relative risk of adenocarcinoma and squamous cell carcinoma of the oesophagus per 5 kg/m^2^ increase in BMI, in various subgroups, excluding the first 5 years of follow-up. RR stratified by year of birth and year of recruitment; adjusted for (where appropriate) joint smoking and alcohol status, OC use, MHT use, exercise, parity, menopausal status and age, age at menarche, region and socioeconomic status. RR, relative risk; CI, confidence interval; BMI, body mass index; OC, oral contraceptive; MHT, menopausal hormone therapy

Analyses of summary trends in risk of each subtype within subgroups defined by joint smoking and alcohol status, and eight other personal characteristics, are shown in Figure 4. There was generally little evidence of any material variation in BMI-associated risks by any of the characteristics. However the BMI-associated risks for squamous cell carcinoma were slightly stronger in current smokers, as noted previously, and the BMI-associated risks for adenocarcinoma were slightly greater for those aged <70 years than those aged 70+ years (*P*_heterogeneity_=0.009) (Figure 4).

Analyses restricted to adenocarcinoma not otherwise specified (NOS) (ICD-O 81403) yielded similar findings (RR per 5 kg/m^2^ = 1.58, 95% CI = 1.46–1.70, based on 1085 cases), as did analyses restricted to adenocarcinoma of the gastro-oesophageal junction (ICD-10 C160) (RR per 5 kg/m^2^ = 1.38, 95% CI = 1.24–1.53, based on 568 cases). There were very few (33) cases of squamous cell carcinoma of the gastro-oesophageal junction but there was some evidence of a reduced risk per 5 kg/m^2^ increase in BMI (RR = 0.51, 95% CI = 0.30–0.86).

Based on BMI reported at the survey in median year 2006, the estimated BMI-associated risk of adenocarcinoma of 1.53 (95% CI = 1.35–1.73) per 5 kg/m^2^ was somewhat attenuated after adjustment for self-reported factors relating to GORD (heartburn, use of Losec/Zoton and difficulty swallowing) (RR = 1.38, 95% CI = 1.22–1.56); this change was mainly driven by adjustment for self-reported heartburn.

Abdominal adiposity analyses were based on 478 769 women who provided information at the survey in median year 2001 on BMI, WC and WHR, of whom 351 and 605 developed adenocarcinoma and squamous cell carcinoma, respectively, over 5 years later. The RRs per 1-SD of each adiposity index were broadly similar (Table 2). In analyses using the residual method to adjust for BMI, neither WC nor WHR were independently associated with adenocarcinoma risk (*P*_trend_ = 0.3 and *P*_trend_ = 0.06 for WC and WHR, respectively), but WC and WHR were associated with squamous cell carcinoma (*P*_trend_<0.001 for WC and *P*_trend_ = 0.005 for WHR) (Table 2). There were insufficient cases diagnosed 5 or more years after baseline to reliably assess interactions with smoking and alcohol in associations of abdominal adiposity with risk of either subtype.

**Table 2:** Relative risk of adenocarcinoma and squamous cell carcinoma of the oesophagus by abdominal adiposity measures, excluding the first 5 years of follow-up

Measure	Adenocarcinoma		Squamous cell carcinoma	
	No. of cases	RR [95% (gs)CI]	No. of cases	RR [95% (gs)CI]
RR^1^ per 1-SD BMI	351	1.55 (1.35–1.78)	605	0.64 (0.57–0.72)
RR^1^ per 1-SD WC	351	1.46 (1.25–1.71)	605	0.59 (0.51–0.68)
RR^1^ per 1-SD WHR	351	1.52 (1.19–1.94)	605	0.63 (0.52–0.76)
				
RR^2^ by quartile of WC residuals				
1	79	1.00 (0.80–1.25)	190	1.00 (0.87–1.15)
2	86	1.27 (1.02–1.57)	161	0.81 (0.69–0.94)
3	91	1.32 (1.08–1.63)	149	0.78 (0.66–0.92)
4	95	1.17 (0.96–1.44)	105	0.61 (0.50–0.74)
*P* for trend		0.3		<0.001
				
RR^2^ by quartile of WHR residuals				
1	80	1.00 (0.80–1.25)	183	1.00 (0.86–1.16)
2	84	1.16 (0.93–1.44)	156	0.82 (0.70–0.97)
3	75	1.01 (0.80–1.26)	137	0.76 (0.65–0.90)
4	112	1.38 (1.15–1.67)	129	0.74 (0.62–0.88)
*P* for trend		0.06		0.005

RR, relative risk; (gs)CI, (group-specific) confidence interval; BMI, body mass index; WC, waist circumference; WHR, waist-to-hip ratio; SD, standard deviation.

RR^1^ (95% CI), stratified by year of birth and year of recruitment and adjusted for joint smoking and alcohol status, oral contraceptive use, menopausal hormone therapy use, exercise, parity, menopausal status and age, age at menarche, region and socioeconomic status.

RR^2^ (95% gsCI), additionally adjusted for standardized BMI quartiles.

## Discussion

In this large cohort of UK women, we found a marked positive association of BMI with adenocarcinoma of the oesophagus and a similar inverse association of BMI with squamous cell carcinoma. These associations were broadly consistent across follow-up periods and were also evident in never smokers and non-drinkers. Greater WC and WHR appeared to be associated with a reduction in squamous cell carcinoma risk independently of BMI, but had little additional effect on the risk of adenocarcinoma.

Findings from prospective studies have consistently reported increased risks of oesophageal adenocarcinoma ranging from 2- to 4-fold for those in the highest category of BMI compared with those of ‘normal’ BMI,^5^^–^^9^^,^^11^^,^^24^ but these have generally been based on small numbers of cases, especially among women. After taking account of potential reverse causation, which could lead to underestimation of the effect of BMI, we observed a 2-fold increased risk in women of BMI 30+ compared with BMI 22.5–<25 kg/m^2^ (RR = 2.29, 95% CI = 1.94–2.70), based on more than 10 times as many cases in women as in any previous cohort.

There is comparatively little evidence regarding the association of BMI with squamous cell carcinoma in European populations,^5^^,^^7^^,^^11^^,^^24^ although several small studies have reported an inverse association. The largest study in women^5^ reported a 57% risk reduction associated with BMI ≥30 kg/m^2^ compared with BMI 18.5–24.9  kg/m^2^ (RR = 0.43, 95% CI = 0.32–0.59; *P*_trend_ <0.001) but did not adjust for key confounders such as smoking and alcohol intake. Some large Asian studies also found substantial inverse associations between BMI and oesophageal cancer,^10^^,^^12^^,^^25^ the vast majority of which are likely to have been squamous cell carcinomas.^2^ These studies, which were unable to take full account of reverse causation or residual confounding, reported somewhat smaller reductions in risk; however, cases in the Asian studies are likely to have included a small proportion of adenocarcinoma, for which risk is increased with increasing BMI. The causal relevance of any apparent inverse association of BMI with squamous cell carcinoma in previous studies has been hitherto unclear, since few had sufficient power or follow-up to rule out substantial bias from residual confounding due to smoking, or reverse causation due to the effect of early symptoms on weight. Two Chinese prospective studies reported inverse associations in never smokers,^10^^,^^25^ and the only previous study to assess the impact of excluding at least 5 years of follow-up reported a 50–60% reduction in risk per 5 kg/m^2^ but did not adjust for major confounders.^5^ In contrast, we demonstrated a clear reduction in squamous cell carcinoma risk with increasing BMI after adjustment for all likely confounders, in subgroups of women defined by both smoking and alcohol status, and after exclusion of up to 15 years of follow-up, thus providing the strongest evidence to date for a causal association.

Studies of abdominal adiposity (namely WC and WHR) have tended to report positive associations with adenocarcinoma risk,^8^^,^^11^^,^^24^^,^^26^^–^^28^ though few have demonstrated associations specifically in women.^24^ When overall adiposity was taken into account (via adjustment for BMI or hip circumference), these associations remained strong.^8^^,^^11^^,^^24^^,^^27^ Previous studies of abdominal adiposity and squamous cell carcinoma in Western populations have included <200 cases in total, with even fewer among women, and found little evidence of an association,^11^^,^^24^^,^^26^^,^^27^ whereas two Asian studies^10^^,^^29^ reported inverse associations for WC after adjustment for non-adiposity related factors, one of which reported a positive association with WC after additional adjustment for BMI.^29^ Given the high correlation between indices of abdominal and total adiposity, it is difficult to interpret associations of each index with risk, after mutual adjustment for other indices. Our residual-based analyses should minimize such biases, and the findings indicate little additional association of abdominal adiposity with adenocarcinoma risk after controlling for BMI, but do suggest that abdominal adiposity may be particularly important in determining squamous cell carcinoma risk (Table 2).

The association of BMI with oesophageal adenocarcinoma risk is thought to be largely (but not wholly^30^^,^^31^) due to the intermediate association with gastro-oesophageal reflux (GORD). Although abdominal adiposity is considered to be a bigger risk factor for GORD than overall adiposity,^30^^,^^32^ neither WC nor WHR appeared to be associated with increased adenocarcinoma risk in these data, after accounting for BMI. Given that the association between BMI and adenocarcinoma was only slightly attenuated after adjustment for reflux symptoms, it is also possible that the effect of BMI is not entirely due to its effect on GORD, although it may also be that we were unable to adequately capture and adjust for previous exposure to GORD. It has been posited that adiposity may affect adenocarcinoma risk through other pathways, such as inflammation or insulin-like growth factor 1 (IGF1),^33–35^ but direct evidence for such mechanisms remains limited.

Although these data provide convincing evidence for an inverse association between BMI and squamous cell carcinoma, the reasons for such an association are unknown. The relatively high squamous cell carcinoma rates among those with low BMI, particularly in Asian populations, have been attributed to malnutrition,^36^^,^^37^ although there is limited evidence for a role of dietary factors in squamous cell carcinoma risk,^14^^,^^38^ and the approximately linear nature of the observed association does not appear to support this hypothesis. A previous study of squamous cell carcinoma of the head and neck reported greater associations of smoking and/or alcohol intake with risk in those with low BMI,^39^ leading to an apparent inverse association of BMI with risk of such cancers in populations which included smokers. Our finding, however, of an inverse association of BMI with squamous cell carcinoma risk in women who were never smokers and non-drinkers indicates that BMI is not merely attenuating the effects of smoking and alcohol. The greater inverse associations of BMI with squamous cell carcinoma observed here for past and current smokers could be due, at least in part, to imperfect adjustment for smoking patterns in smokers. Although empirical evidence for specific mechanisms is sparse, estrogen has been hypothesized to protect against squamous cell carcinoma,^40^ which would be consistent with our findings, given that adiposity is associated with increased serum estradiol levels after the menopause.^41^^,^^42^

To our knowledge, this is the largest prospective study of adiposity and oesophageal subtypes in a European population. The range of exposure information available allowed for adjustment for all known confounders, as well as assessment of the potential for residual confounding by smoking and other risk factors. The large number of cases and extremely long follow-up meant that we could exclude the possibility that the observed associations were due to reverse causation. Although we used self-reported measures to assign women to categories of each anthropometric measure at baseline, a validation study in ∼4000 participants^18^ found good agreement between self-reported measures at baseline and those measured by clinicians up to 9 years later, and estimated trends were also corrected for measurement error by using these measured values. Although our findings suggest that the effect of BMI on adenocarcinoma risk is not entirely mediated by GORD, we did not have detailed information on GORD symptoms, and it is possible that adjustment for severity or duration of GORD could have resulted in greater attenuation of BMI-associated risks. A further limitation of our study is that it is restricted to European women, and as such the findings are not necessarily generalizable to other populations.

## Conclusion

In this comprehensive study of adiposity and the two main histological subtypes of oesophageal cancer in middle-aged UK women, we found robust evidence that greater BMI increases the risk of adenocarcinoma and decreases the risk of squamous cell carcinoma. Further work is needed to understand the mechanisms underlying the opposite effects of adiposity on these cancer subtypes.

## Membership of the Million Women Study

Advisory committee: Emily Banks, Lucy Carpenter, Carol Dezateux (chair), Sarah Floud, Jane Green, Julietta Patnick, Richard Peto, Gillian Reeves, Cathie Sudlow. Coordinating centre staff: Simon Abbott, Rupert Alison, Sarah Atkinson, Krys Baker, Angela Balkwill, Isobel Barnes, Judith Black, Roger Blanks, Anna Brown, Andrew Chadwick, Dave Ewart, Sarah Floud, Kezia Gaitskell, Toral Gathani, Laura Gerrard, Adrian Goodill, Carol Hermon, Isobel Lingard, Sau Wan Kan, Nicky Langston, Keren Papier, Kirstin Pirie, Gillian Reeves, Keith Shaw, Emma Sherman, Karl Smith-Byrne, Helena Strange, Siân Sweetland, Ruth Travis, Lyndsey Trickett, Clare Wotton, Owen Yang, Heather Young.

## Ethics approval

Ethical approval was provided by the Oxford and Anglia Multi-Centre Research Ethics Committee (97/5/001).

## Supplementary Material

dyad094_Supplementary_Data

## Data Availability

Anonymized data used here can be accessed by application to the investigators and to the providers of follow-up data (e.g. NHS Digital) from any qualified investigator. The Million Women Study Data Access Policy can be viewed at [millionwomenstudy.org/data_access].

## References

[1] Sung H , Ferlay J, Siegel RL et al Global Cancer Statistics 2020: GLOBOCAN estimates of incidence and mortality worldwide for 36 cancers in 185 countries. CA Cancer J Clin 2021;71:209–49. 33538338 10.3322/caac.21660

[2] Arnold M , Ferlay J, van Berge Henegouwen MI et al Global burden of oesophageal and gastric cancer by histology and subsite in 2018. Gut 2020;69:1564–71. 32606208 10.1136/gutjnl-2020-321600

[3] Offman J , Pesola F, Sasieni P. Trends and projections in adenocarcinoma and squamous cell carcinoma of the oesophagus in England from 1971 to 2037. Br J Cancer 2018;118:1391–98. 29563637 10.1038/s41416-018-0047-4 PMC5959941

[4] NHS. *NHS Cancer Data - Get Data Out 2022*. https://www.cancerdata.nhs.uk/getdataout/oes (17 May 2022, date last accessed).

[5] Engeland A , Tretli S, Bjorge T. Height and body mass index in relation to esophageal cancer; 23-year follow-up of two million Norwegian men and women. Cancer Causes Control 2004;15:837–43. 15456997 10.1023/B:CACO.0000043434.21558.ea

[6] Merry AH , Schouten LJ, Goldbohm RA et al Body mass index, height and risk of adenocarcinoma of the oesophagus and gastric cardia: a prospective cohort study. Gut 2007;56:1503–11. 17337464 10.1136/gut.2006.116665 PMC2095659

[7] Lindkvist B , Johansen D, Stocks T et al Metabolic risk factors for esophageal squamous cell carcinoma and adenocarcinoma: a prospective study of 580,000 subjects within the Me-Can project. BMC Cancer 2014;14:103. 24548688 10.1186/1471-2407-14-103 PMC3929907

[8] O'Doherty MG , Freedman ND, Hollenbeck AR et al A prospective cohort study of obesity and risk of oesophageal and gastric adenocarcinoma in the NIH-AARP Diet and Health Study. Gut 2012;61:1261–68. 22174193 10.1136/gutjnl-2011-300551 PMC3504700

[9] Lindblad M , Rodriguez LA, Lagergren J. Body mass, tobacco and alcohol and risk of esophageal, gastric cardia, and gastric non-cardia adenocarcinoma among men and women in a nested case-control study. Cancer Causes Control 2005;16:285–94. 15947880 10.1007/s10552-004-3485-7

[10] Wang L , Jin G, Yu C et al; China Kadoorie Biobank Collaborative Group. Cancer incidence in relation to body fatness among 0.5 million men and women: Findings from the China Kadoorie Biobank. Int J Cancer 2020;146:987–98. 31115907 10.1002/ijc.32394 PMC7614994

[11] Sanikini H , Muller DC, Chadeau-Hyam M et al Anthropometry, body fat composition and reproductive factors and risk of oesophageal and gastric cancer by subtype and subsite in the UK Biobank cohort. PLoS One 2020;15:e0240413. 33079929 10.1371/journal.pone.0240413 PMC7575071

[12] Tran GD , Sun XD, Abnet CC et al Prospective study of risk factors for esophageal and gastric cancers in the Linxian general population trial cohort in China. Int J Cancer 2005;113:456–63. 15455378 10.1002/ijc.20616

[13] Green J , Reeves GK, Floud S et al; Million Women Study Collaborators. Cohort Profile: The Million Women Study. Int J Epidemiol 2019;48:28–29e. 29873753 10.1093/ije/dyy065 PMC6380310

[14] Xie SH , Lagergren J. Risk factors for oesophageal cancer. Best Pract Res Clin Gastroenterol 2018;36-37:3–8. 30551854 10.1016/j.bpg.2018.11.008

[15] World Health Organization. International Statistical Classification of Diseases and Related Health Problems - Tenth Revision. Geneva: WHO, 1992. 3376487

[16] Percy VH , Muir C. International Classification of Diseases for Oncology: ICD-O. 2nd edn. Geneva: WHO, 1990.

[17] Fritz P , Jack A. International Classification of Diseases for Oncology: ICD-O. 3rd edn. Geneva: WHO, 2000.

[18] Wright FL , Green J, Reeves G et al; Million Women Study Collaborators. Validity over time of self-reported anthropometric variables during follow-up of a large cohort of UK women. BMC Med Res Methodol 2015;15:81. 26450616 10.1186/s12874-015-0075-1 PMC4599695

[19] Castellsagué X , Muñoz N, De Stefani E et al Independent and joint effects of tobacco smoking and alcohol drinking on the risk of esophageal cancer in men and women. Int J Cancer 1999;82:657–64. 10417762 10.1002/(sici)1097-0215(19990827)82:5<657::aid-ijc7>3.0.co;2-c

[20] Allen NE , Beral V, Casabonne D et al; Million Women Study Collaborators. Moderate alcohol intake and cancer incidence in women. J Natl Cancer Inst 2009;101:296–305. 19244173 10.1093/jnci/djn514

[21] Easton DF , Peto J, Babiker AG. Floating absolute risk: an alternative to relative risk in survival and case-control analysis avoiding an arbitrary reference group. Stat Med 1991;10:1025–35. 1652152 10.1002/sim.4780100703

[22] Plummer M. Improved estimates of floating absolute risk. Stat Med 2004;23:93–104. 14695642 10.1002/sim.1485

[23] Benetou V , Bamia C, Trichopoulos D et al Associations of anthropometric characteristics with blood cholesterol fractions among adults. The Greek EPIC study. Eur J Clin Nutr 2006;60:942–48. 16465197 10.1038/sj.ejcn.1602403

[24] Sanikini H , Muller DC, Sophiea M et al Anthropometric and reproductive factors and risk of esophageal and gastric cancer by subtype and subsite: Results from the European Prospective Investigation into Cancer and Nutrition (EPIC) cohort. Int J Cancer 2020;146:929–42. 31050823 10.1002/ijc.32386 PMC6973006

[25] Smith M , Zhou M, Whitlock G et al Esophageal cancer and body mass index: results from a prospective study of 220,000 men in China and a meta-analysis of published studies. Int J Cancer 2008;122:1604–10. 18059032 10.1002/ijc.23198

[26] Lin Y , Ness-Jensen E, Hveem K et al Metabolic syndrome and esophageal and gastric cancer. Cancer Causes Control 2015;26:1825–34. 26450604 10.1007/s10552-015-0675-4

[27] Corley DA , Kubo A, Zhao W. Abdominal obesity and the risk of esophageal and gastric cardia carcinomas. Cancer Epidemiol Biomarkers Prev 2008;17:352–58. 18268119 10.1158/1055-9965.EPI-07-0748 PMC2670999

[28] MacInnis RJ , English DR, Hopper JL et al Body size and composition and the risk of gastric and oesophageal adenocarcinoma. Int J Cancer 2006;118:2628–31. 16353151 10.1002/ijc.21638

[29] Cho JH , Shin CM, Han KD et al Abdominal obesity increases risk for esophageal cancer: a nationwide population-based cohort study of South Korea. J Gastroenterol 2020;55:307–16. 31792601 10.1007/s00535-019-01648-9

[30] Lagergren J. Influence of obesity on the risk of esophageal disorders. Nat Rev Gastroenterol Hepatol 2011;8:340–47. 21643038 10.1038/nrgastro.2011.73

[31] Hoyo C , Cook MB, Kamangar F et al Body mass index in relation to oesophageal and oesophagogastric junction adenocarcinomas: a pooled analysis from the International BEACON Consortium. Int J Epidemiol 2012;41:1706–18. 23148106 10.1093/ije/dys176 PMC3535758

[32] Lagergren J. Controversies surrounding body mass, reflux, and risk of oesophageal adenocarcinoma. Lancet Oncol 2006;7:347–49. 16574550 10.1016/S1470-2045(06)70660-X

[33] Cook MB , Barnett MJ, Bock CH et al Prediagnostic circulating markers of inflammation and risk of oesophageal adenocarcinoma: a study within the National Cancer Institute Cohort Consortium. Gut 2019;68:960–68. 30121626 10.1136/gutjnl-2018-316678 PMC6379150

[34] Singh S , Sharma AN, Murad MH et al Central adiposity is associated with increased risk of esophageal inflammation, metaplasia, and adenocarcinoma: a systematic review and meta-analysis. Clin Gastroenterol Hepatol 2013;11:1399–412.e7. 23707461 10.1016/j.cgh.2013.05.009 PMC3873801

[35] Calle EE , Kaaks R. Overweight, obesity and cancer: epidemiological evidence and proposed mechanisms. Nat Rev Cancer 2004;4:579–91. 15286738 10.1038/nrc1408

[36] Gao YT , McLaughlin JK, Gridley G et al Risk factors for esophageal cancer in Shanghai, China. II. Role of diet and nutrients. Int J Cancer 1994;58:197–202. doi: 10.1002/ijc.2910580209 [published Online First: 1994/07/15] 8026881

[37] Samanic C , Gridley G, Chow WH et al Obesity and cancer risk among white and black United States veterans. Cancer Causes Control 2004;15:35–43. 14970733 10.1023/B:CACO.0000016573.79453.ba

[38] World Cancer Research Fund/American Institute of Cancer Research. *Continuous Update Report: Diet, Nutrition, Physical Activity and Oesophageal Cancer*. 2016. https://www.wcrf.org/oesophageal-cancer-2016 (30th March 2021, date last accessed).

[39] Gaudet MM , Olshan AF, Chuang SC et al Body mass index and risk of head and neck cancer in a pooled analysis of case-control studies in the International Head and Neck Cancer Epidemiology (INHANCE) Consortium. Int J Epidemiol 2010;39:1091–102. 20123951 10.1093/ije/dyp380 PMC2929351

[40] Chandanos E , Lagergren J. The mystery of male dominance in oesophageal cancer and the potential protective role of oestrogen. Eur J Cancer 2009;45:3149–55. 19804965 10.1016/j.ejca.2009.09.001

[41] Grodin JM , Siiteri PK, MacDonald PC. Source of estrogen production in postmenopausal women. J Clin Endocrinol Metab 1973;36:207–14. 4688315 10.1210/jcem-36-2-207

[42] Key TJ , Appleby PN, Reeves GK et al; Endogenous Hormones and Breast Cancer Collaborative Group. Circulating sex hormones and breast cancer risk factors in postmenopausal women: reanalysis of 13 studies. Br J Cancer 2011;105:709–22. 21772329 10.1038/bjc.2011.254 PMC3188939

