# Supplementary material for: Adiposity and risk of oesophageal cancer subtypes in the Million Women Study
Source: Int J Epidemiol. 2023 Jul 12;52(6):1795–804. doi: 10.1093/ije/dyad094 (PMC10749780; doi:10.1093/ije/dyad094)
Supplement: dyad094_Supplementary_Data [file dyad094_supplementary_data.docx]

**SUPPLEMENTARY MATERIALS**

**Table of Contents Page**

Supplementary Methods…………………………………………………………………………........2

References…………………………………………………………………………..............................7

Supplementary Table S1………………………………………………………………………….........8

Supplementary Figure S1…………………………………………………………………………........9

**Supplementary Methods**

*Outcome and exposure assessment*

Classification of oesophageal cancers by subtype was based on cancers with ICD-10 code C15, with ICD-O morphology coding as given in the table below:

| Adenocarcinoma | Squamous Cell Carcinoma |
| --- | --- |
| 8140-8145  8190-8231  8260-8263  8310  8315  8480-8490  8570-8575 | 8050-8086 |

Participants were asked to report their current height and weight at the recruitment questionnaire in median year 1998, and were asked again about weight at subsequent resurveys. BMI was calculated as current weight (in kg) divided by the square of their height in metres (m^2^), using self-reported weight at the relevant baseline survey and self-reported height at recruitment. Valid measures ranged from 1.2-2.0m for height and 35-150kg for weight. Waist and hip measurements were taken from the survey in median year 2001: waist-hip-ratio (WHR) was calculated as waist circumference (cm) divided by hip circumference (cm). A previously published validation study in ~4000 randomly selected Million Women Study participants found clinical measurements of anthropometric variables obtained in median year 2008 to be very strongly, or moderately strongly, correlated with corresponding self-reported measures recorded over a 9 year period^1^; Pearson correlation between self-reported and measured values ranged between 0.85 and 0.95 for weight, height and BMI, and between 0.66 and 0.82 for waist and hip circumference.

*Statistical analyses*

Analyses with the recruitment questionnaire in median year 1998 as baseline were stratified by year of birth and year of recruitment, and adjusted for region, socioeconomic status, joint alcohol and smoking status, use of oral contraceptives, use of menopausal hormone therapy, menopausal status and age, strenuous exercise, number of full-term pregnancies, and age at menarche, with categories as given in the table below:

| Variable | Categories |
| --- | --- |
| Stratification:  Year of birth  Year of recruitment | ≤1935, 1936-1940, 1941-1945, ≥1946  1996, 1997, 1998, 1999, ≥2000 |
| Adjustment:  Region  Socioeconomic status  Alcohol & Smoking      Use of oral contraceptives  Use of menopausal hormone therapy  Strenuous exercise  Number of full-term pregnancies  Age at menarche  Menopausal status and age | 10 regions of cancer registries at recruitment  Quintiles of deprivation based on the Townsend index^2^  18 categories based on cross classification of women according to smoking status (never, past, never/past, current: 1-9/10-14/15+ cigarettes per day) with alcohol intake (non-drinker/1-6/7+ drinks per week)  Never, ever  Never, past, current  ≤once, ≥twice per week  0, 1, 2, 3, 4+  <13, 13-14, 15+  Premenopausal/perimenopausal, postmenopausal with known age <47/47-49/50-51/52+, postmenopausal with unknown age, unknown menopausal status |

For each adjustment variable, women with missing values were assigned to a separate category. Missing data accounted for <4% for each covariate apart from menopausal status (15.5%).

Analyses with the questionnaire in median year 2001 as baseline were stratified by year of birth and year of baseline questionnaire, and adjusted for alcohol, smoking, and their interaction, use of menopausal hormone therapy, and menopausal status and age, which were all taken from baseline questionnaire, as well as region, socioeconomic status, use of oral contraceptives, strenuous exercise, number of full-term pregnancies and age at menarche which were all taken from the recruitment questionnaire. Categories for all these variables are given in the table below:

| Variable | Categories |
| --- | --- |
| Stratification:  Year of birth  Year of first resurvey | ≤1935, 1936-1940, 1941-1945, ≥1946  <2000, 2000, 2001, 2002, 2003, ≥2004 |
| Adjustment:  Region  Socioeconomic status  Alcohol & Smoking      Use of oral contraceptives  Use of menopausal hormone therapy  Strenuous exercise  Number of full-term pregnancies  Age at menarche  Menopausal status | 10 regions of cancer registries  Quintiles of Townsend deprivation^2^  15 categories based on cross classification of women according to smoking status (never, past, current: 1-9/10-14/15+ cigarettes per day) with alcohol intake (non-drinker/1-6/7+ drinks per week)  Never, ever  Never, past, current, ever (status nk)  ≤once, ≥twice per week  0, 1, 2, 3, 4+  <13, 13-14, 15+  Premenopausal/perimenopausal, postmenopausal with known age <47/47-49/50-51/52+, postmenopausal with unknown age, menopausal status unknown |

*Sensitivity analyses*

A sensitivity analysis was carried out to investigate the effect of restricting adenocarcinoma diagnoses to those with ICD-O morphology code 81403, corresponding to adenocarcinoma not otherwise specified (NOS), in order to assess any influential effect of specified types of adenocarcinoma. We also examined the association of BMI with adenocarcinoma of the gastroesophageal junction (ICD-10 C160), as it can be difficult to distinguish cancers in the lower oesophagus and gastric cardia (almost all of which are adenocarcinoma) leading to potential site misclassification^3 4^.

Information on treatment for, and symptoms of, gastro-oesophageal reflux (GORD) were first asked about at the survey in median year 2006, at which women were asked: “Have you taken Losec/Zoton for most of the last four weeks?”, and “How often are you troubled by heartburn/difficulty swallowing?” (possible responses: never/rarely, less than once a week, about once a week, and more than once a week). A sensitivity analysis was conducted in which variables representing these questions were added to a fully adjusted model examining the relationship of BMI reported at the same survey with risk of adenocarcinoma of the oesophagus, excluding the first 5 years of follow-up.

*Analyses of abdominal adiposity*

To allow direct comparisons of the magnitude of the associations with different indices of adiposity, taking account of possibly differing measurement error in each index, trends were first assessed by categorising women according to standardised measures (subtracting the mean and dividing by the standard deviation) of BMI, waist circumference (WC) and waist-hip-ratio (WHR), creating quartiles of these measures and then fitting a scored variable, using the median remeasured value in each quartile (from the sub-study in median year 2008^1^), to estimate the RR per 1SD of each measure. In order to assess the extent to which any associations of abdominal adiposity measures were independent of the effect of overall adiposity, we fitted Cox models accounting for BMI using the residual method^5^. Here, the residuals of a linear regression of WC, and of WHR, on BMI (adjusted for age) were obtained and women were subsequently divided into quartiles on the basis of their residual values. These quartiles were fitted in a Cox model with the standardised BMI variable (and all other adjustments as above). Tests for trend across these quartiles were assessed by scoring the quartiles (1-4) and treating this variable as continuous in the model.

**References**

1. Wright FL, Green J, Reeves G, et al. Validity over time of self-reported anthropometric variables during follow-up of a large cohort of UK women. *BMC Med Res Methodol* 2015;15:81. doi: 10.1186/s12874-015-0075-1 [published Online First: 2015/10/10]

2. Townsend P, Beattie. Health and Deprivation: Inequality and the North. London: Croom Helm 1988.

3. Forman D. Review article: oesophago-gastric adenocarcinoma -- an epidemiological perspective. *Aliment Pharmacol Ther* 2004;20 Suppl 5:55-60; discussion 61-2. doi: 10.1111/j.1365-2036.2004.02133.x [published Online First: 2004/10/01]

4. Lindblad M, Ye W, Lindgren A, et al. Disparities in the classification of esophageal and cardia adenocarcinomas and their influence on reported incidence rates. *Ann Surg* 2006;243(4):479-85. doi: 10.1097/01.sla.0000205825.34452.43 [published Online First: 2006/03/23]

5. Benetou V, Bamia C, Trichopoulos D, et al. Associations of anthropometric characteristics with blood cholesterol fractions among adults. The Greek EPIC study. *Eur J Clin Nutr* 2006;60(8):942-8. doi: 10.1038/sj.ejcn.1602403 [published Online First: 2006/02/09]

**Supplementary Table S1: Relative risk per 5kg/m^2^ increase in BMI for adenocarcinoma and squamous cell carcinoma of the oesophagus after adjustment for various confounders^a^**

|  | | Adenocarcinoma | | |  | Squamous cell carcinoma | | |
| --- | --- | --- | --- | --- | --- | --- | --- | --- |
|  | | RR (95%CI) | χ^2 *^ | %change |  | RR (95%CI) | χ^2 *^ | %change |
|  |  | | | | | | | |
| Stratified by year of birth, year of recruitment, and adjusted for region and socioeconomic status: | | | | | | | | |
|  | | 1.59 (1.48-1.70) | 173 |  |  | 0.55 (0.51-0.59) | 255 |  |
| Independently adjusted for: | | | | | | | | |
| Smoking | | 1.63 (1.52-1.74) | 191 | 10.5% |  | 0.56 (0.52-0.61) | 228 | -10.7% |
| Alcohol | | 1.56 (1.45-1.67) | 158 | -8.3% |  | 0.55 (0.51-0.59) | 248 | -3.0% |
| Joint Smoking and Alcohol status | | 1.59 (1.48-1.70) | 171 | -0.9% |  | 0.57 (0.53-0.61) | 224 | -12.4% |
| OC use | | 1.58 (1.48-1.69) | 171 | -0.9% |  | 0.55 (0.51-0.59) | 256 | 0.4% |
| MHT use | | 1.58 (1.47-1.69) | 169 | -2.0% |  | 0.54 (0.50-0.59) | 260 | 1.8% |
| Menopausal status & age | | 1.59 (1.48-1.70) | 175 | 1.2% |  | 0.55 (0.51-0.59) | 250 | -2.1% |
| Exercise | | 1.57 (1.47-1.69) | 166 | -3.8% |  | 0.55 (0.51-0.59) | 252 | -1.1% |
| Parity | | 1.59 (1.48-1.70) | 174 | 0.8% |  | 0.55 (0.51-0.59) | 249 | -2.4% |
| Age at menarche | | 1.57 (1.46-1.68) | 163 | -5.8% |  | 0.54 (0.50-0.59) | 256 | 0.1% |
|  | |  |  |  |  |  |  |  |
| Adjusted for all covariates | | 1.57 (1.46-1.69) | 157 | -9.4% |  | 0.57 (0.53-0.61) | 214 | -16.2% |
|  | |  |  |  |  |  |  |  |

^a^ RR = relative risk; CI = confidence interval; BMI = body mass index; OC = oral contraceptive; MHT = menopausal hormone therapy

^*^ likelihood-ratio χ^2^ test statistic associated with inclusion of adiposity term in a model with specified adjustments

**Supplementary Figure S2: Flowchart showing exclusion of study participants for these analyses**

1 364 268 women available for analysis

1 324 951 women remaining

39 317 women with previous cancer excluded

1 255 529 women remaining in the analysis dataset

69 422 women with unknown BMI excluded

**Supplementary Figure S2: Relative risk of adenocarcinoma and squamous cell carcinoma of the oesophagus by BMI, in subgroups of smoking status and weekly alcohol intake, excluding the first five years of follow-up**


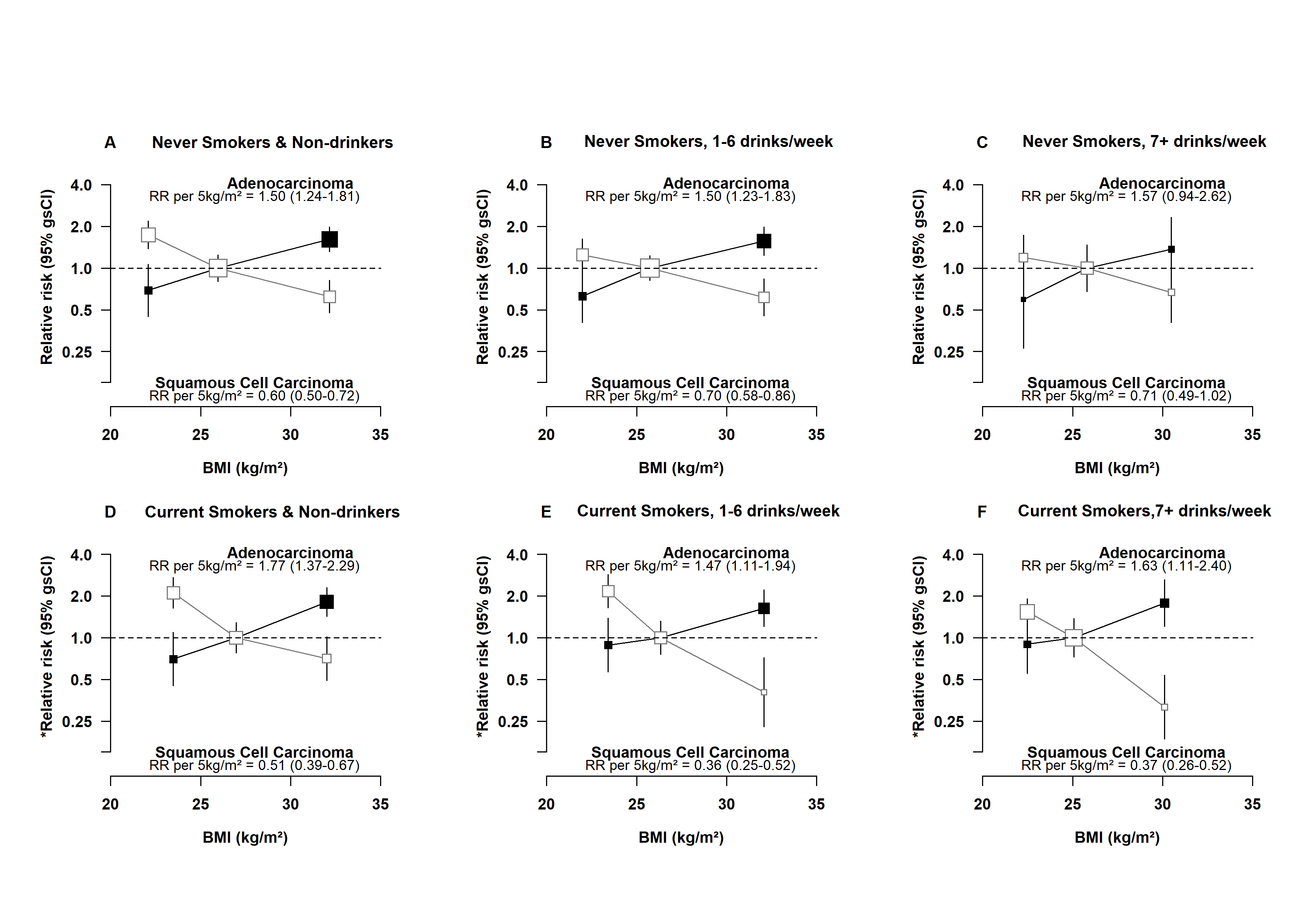


RR = relative risk; (gs)CI = (group specific) confidence interval; BMI = body mass index; OC = oral contraceptive; MHT = menopausal hormone therapy

All analyses are stratified by year of birth and year of recruitment, and adjusted for OC use, MHT use, exercise, parity, menopausal status and age, age at menarche, region and socioeconomic status; * analyses of current smokers were additionally adjusted for joint number of cigarettes smoked per day and alcohol status
